# Supplementary material for: Design and Evaluation of Engineered Extracellular Vesicle (EV)-Based Targeting for EGFR-Overexpressing Tumor Cells Using Monobody Display
Source: Bioengineering (Basel). 2022 Jan 29;9(2):56. doi: 10.3390/bioengineering9020056 (PMC8869414; doi:10.3390/bioengineering9020056)
Supplement: Supplementary file 1 [file bioengineering-09-00056-s001.zip › bioengineering-1541405-supplementary.pdf]

bioengineering

Article

Design and Evaluation of Engineered Extracellular Vesicle (EV)-based Targeting for EGFR-Overexpressing Tumor Cells using Monobody Display

Hiroaki Komuro [1,2], Shakhlo Aminova [1,2], Katherine Lauro [1,2], Daniel Woldring [1,3] and Masako Harada [1,2] *

Supplementary Materials:

(A)

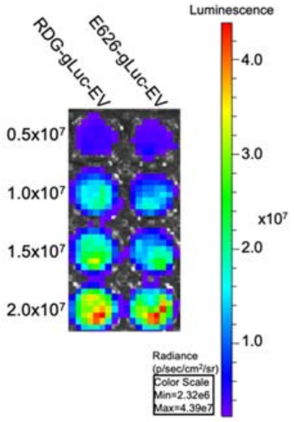

(B)

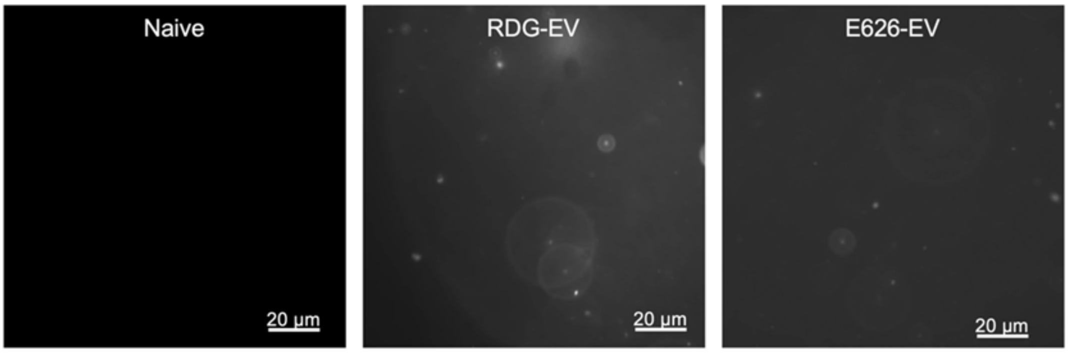

Figure S1. Assessment of EV labeling (A) Bioluminescence radiance measurement of E626-gLuc EVs and RDG-gLuc EVs (**B**) Direct Fluorescence microscopic image of Naïve EVs, E626-mCherry EVs and RDG-mCherry EVs.

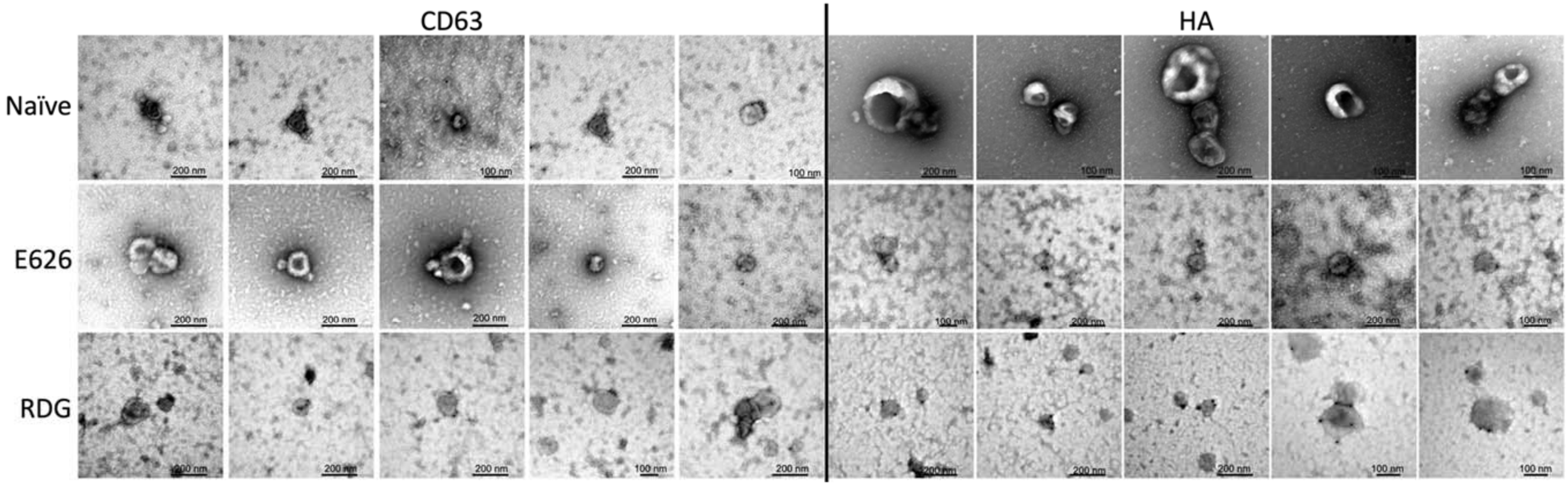

Figure S2. Immuno-Transmission electron microscopy (Immuno-TEM) images of CD63 or HA labeled naïve EVs, E626-EVs and RDG-EVs.

Table S1. The list of DNA sequences used in this study

Primer	Sequence (5'-3')	Template
C1C2-F	GAATGTGTCGAGCCACTGGG	pcS-C1C2 backbone
HA-R	AGCATAATCTGGAACATCATATGG	
**Synthetic DNA Fragments**		
RDG gBlock	GTTCCAGATTATGCTTCCTCCGACTCTCCGCGTAACCTGGAGGTTACCAACGCAACTCCGAA<br>CTCTCTGACTATTTCTTGGGATGCGCCGGCTGTTACCGTACGTTACTACCGTATCACCTACGG<br>CGAAACTGGTGGTAACTCCCCGAGCCAGGAATTCACTGTTCCGGGTTCGCGCTCTACCGCG<br>ACCATCAGCGGTCTGAAACCGGGCCAGGATTATACCATTACCGTGTACGCTGTAACCGGTC<br>GCGACGGCAGCCCGGCGTCCAGCCGTCCAATCAGCATCAATTATCGCACCGAAATCGACAA<br>ACCGTCTCAGGGTGGAGGCGGTTCA	RDG monobody
E626 gBlock	gttccagattatgctAGCAGCGATAGCCCGCGCAACCTGGAAGTGACCAACGCGACCCCGAACA<br>GCCTGACCATTAGCTGGTTTGATTATGCGGTGACCTATTATCGCATTACCTATGGCGAAACC<br>GGCGGCAACAGCCCGAGCCAGGAATTTACCGTGCCGGGCTGGATTAGCACCGCGACCATTA<br>GCGGCCTGAAACCGGGCCAGGATTATACCATTACCGTGTATGCGGTGACCGATAACAGCCA<br>TTGGCCGTTTCGCAGCACCCCGATTAGCATTAACTATCGCACCGAAATTGATAAACCGAGCC<br>AGGGTGGAGGCGGTTCA	E626 monobody
PAS Linker	GGTGGAGGCGGTTCAGGCGGAGGTGGCTCTGGCGGTGGCGGATCGGCCTCTCCAGCTGCA<br>CCTGCTCCAGCAAGCCCTGCTGCACCAGCTCCGTCTGCTCCTGCTGCCTCTCCAGCTGCACCT<br>GCTCCAGCTTCTCCAGCAGCTCCTGCACCTAGTGCTCCTGCTGAATGTGTCGAGCCA	G4S-PAS Linker
